# Retrospective analysis of the influence of 25-hydroxyvitamin D on disease progression and survival in pancreatic cancer

**DOI:** 10.1186/s12937-016-0135-3

**Published:** 2016-02-12

**Authors:** Erica M. McGovern, Mark E. Lewis, Michelle L. Niesley, Nhu Huynh, Jeffrey B. Hoag

**Affiliations:** 1 Department of Clinical Research, Cancer Treatment Centers of America® at Eastern Regional Medical Center, 1331 E. Wyoming Ave, Philadelphia, PA 19124 USA; 2 Department of Nutrition, Cancer Treatment Centers of America® at Eastern Regional Medical Center, 1331 E. Wyoming Ave, Philadelphia, PA 19124 USA; 3 Department of Pulmonary and Critical Care Medicine, Cancer Treatment Centers of America® at Eastern Regional Medical Center, 1331 E. Wyoming Ave, Philadelphia, PA 19124 USA

**Keywords:** Body mass index, Pancreatic neoplasms, Vitamin D deficiency, Overall survival, Progression free survival, Cancer

## Abstract

**Background:**

Vitamin D deficiency is implicated in neoplastic processes in multiple organs, including the pancreas. While animal and human data have established a relationship between serum vitamin D (25(OH)D) and the development of pancreatic cancer, few studies have examined the effects of 25(OH)D on time to progression (TTP) and overall survival (OS) in this patient population. We hypothesize that lower baseline serum concentrations (BSC) of 25(OH)D will be associated with decreased TTP and OS.

**Methods:**

This retrospective analysis of 1222 patients with pancreatic cancer aims to identify potential relationships between 25(OH)D and both TTP and OS, while controlling for the effects of ethnicity and body mass index (BMI). Baseline 25(OH)D was divided into quartiles defined as deficient (<20 ng/mL), insufficient (20–39 ng/mL), sufficient (40–59 ng/mL), and optimal (≥60 ng/ml). Statistical significance was declared if the two-sided *p*-value was ≤ 0.05.

**Results:**

For the 627 subjects included for analysis, the median 25(OH)D was 27 ng/mL (range 4 to 114), 30.0 % were 25(OH)D deficient (<20 ng/mL), and 47.2 % were insufficient (20–39 ng/mL). Ethnicity (*p* < 0.0001) and BMI (*p* = 0.05) were significantly associated with (BSC)of 25(OH)D, while TTP (*p* = 0.39) and OS (*p* = 0.37) were not associated.

**Conclusion:**

Suboptimal vitamin D levels (<60 ng/mL) occurred in 96 % of patients analyzed. Both ethnicity and BMI were statistically significantly associated with vitamin D deficiency and insufficiency. Similar to results previously reported in the literature, this analysis did not identify a significant association between BSC of 25(OH)D and OS or TTP in patients with pancreatic cancer.

## Background

Vitamin D is a steroid hormone primarily obtained through the synthesis of 7-dehydrocholesterol following sun exposure, and secondarily through dietary intake of certain fish, fortified foods and beverages, and dietary supplements. The optimal serum concentration of 25-hydroxyvitamin D (25(OH)D) varies among sources. A widely accepted ideal level is 35–55 ng/mL, but other sources site a broader range of 30–70 ng/mL as optimal [1–3]. The National Health and Nutrition Examination Survey (2005 to 2006) identified a 41.6 % prevalence of vitamin D deficiency (serum 25(OH)D <20 ng/mL) in the United States [4]. Vitamin D levels are affected by ethnicity, body mass index (BMI), geographic exposure to sunlight, age, and disease [3]. Because of the vast biological role of vitamin D, especially in mechanisms commonly associated with cancer, understanding the effects of vitamin D deficiency on disease development and progression is critical.

Although the biologically active form of vitamin D is 1,25-hydroxyvitamin D (1,25(OH)_2_D), total circulating vitamin D from dietary sources, supplements, and sun exposure are best represented by 25(OH)D [5, 6]. Not only is vitamin D responsible for regulating calcium and phosphorous levels in the human body, but it also has anti-proliferative and immunomodulatory effects via autocrine and paracrine signaling [1]. As a lipid-soluble molecule, it readily diffuses across plasma membranes. By binding to the vitamin D receptor (VDR) on the nucleus, it affects target genes involved in intracellular signaling pathways including cell growth, differentiation, adhesion, and apoptosis, making it of interest to study in relation to cancer [5, 6]. Alteration of such cellular mechanisms plays a critical role in cancer development and suggests a potential relationship between vitamin D and cancer.

While the relationship between serum 25(OH)D levels and cancer opens a broad area for analysis, ample research has been conducted analyzing vitamin D and risk of cancer development in prostate, breast, and colorectal cancers [3, 6–10]. Vitamin D supplements have been shown to reduce the risk of cancer development by 77 % when compared to and verified with placebos, and a 2006 study observed a 29 % reduction in cancer death rate for every 10 ng/mL increase in vitamin D [11]. Similarly, ambient exposure to ultraviolet light, especially ultraviolet B-rays (UVB), has been shown to reduce the risk of pancreatic cancer development [12–14]. Despite the evidence supporting decreased risk of pancreatic cancer in individuals with higher serum 25(OH)D, the relationship between serum 25(OH)D and its effect on pancreatic cancer progression has not been extensively studied. Pancreatic cancer is the fourth leading cause of cancer related death in the United States with a 6 % 5-year survival rate, and 75 % mortality rate within the first year of diagnosis [7, 15, 16]. Contradictory results describing the relationship between vitamin D and pancreatic cancer make it difficult for researchers to draw consistent conclusions [17, 18]. While multiple studies suggest adequate 25(OH)D levels may decrease incidence of pancreatic cancer, Stolzenberg et al. conducted a pooled, nested, case–control study of eight cohorts, finding that higher levels of vitamin D (>40 ng/mL) were associated with a two-fold increase in risk of pancreatic cancer development (OR = 2.12, 95 % CI: 1.23, 3.64) [17]. Such results suggest vitamin D supplementation in cancer patients should be monitored carefully.

Despite this data, in vitro and in vivo studies of pancreatic cancer cell lines found that 25(OH)D inhibited cell line growth, while analogs of 25(OH)D inhibited pancreatic cell proliferation, induced differentiation, promoted apoptosis in vitro, and inhibited pancreatic tumor growth in vivo [18–20]. A study published in May 2015 found that activation of vitamin D/VDR signaling led to inhibition of FOXM1, a direct transcriptional target of VDR, causing a suppression of tumor stemness, growth, and metastasis [15]. These results imply vitamin D possesses anti-tumorigenic properties that may be useful in preventing development or progression of cancer.

Although in vitro studies have identified vitamin D and its receptor as potential targets for inhibiting pancreatic cancer development or progression, genetic studies to date have not identified an association between the 25(OH)D, the VDR, and pancreatic cancer [21, 22]. In an analysis of 11 genes (213 single nucleotide polymorphisms) related to vitamin D synthesis, metabolism, and signaling, no individual genes were significantly associated with pancreatic cancer [22].

The first study (CALGB 151006) to examine the relationship between serum 25(OH)D in patients with pancreatic cancer and time to progression (TTP) and overall survival (OS) was a correlative study conducted in May 2014 [23]. These results did not support a statistically significant relationship between 25(OH)D levels and TTP or OS [23]. This current retrospective study aims to contribute to the literature exploring the relationship between vitamin D and TTP and OS in pancreatic cancer patients. It is hypothesized that lower baseline serum concentrations (BSC) of 25(OH)D will be associated with decreased TTP and OS.

## Methods

### Study population

Eligible participants included patients with pancreatic cancer age 18 years or older, seen at any one of the five Cancer Treatment Centers of America (CTCA) hospitals between January 1, 2011, and April 30, 2014, who had one or more recorded serum 25(OH)D blood draws. Patients were excluded from the study if there was a currently active second malignancy other than non-melanomatous skin cancer or carcinoma in-situ of cervix. Institutional Review Board (IRB) exemption was obtained for this project. For each study participant, data points collected included but were not limited to demographics, number of treatment regimens prior to presentation at CTCA, stage of pancreatic cancer, and value of each serum 25(OH)D draw.

### Vitamin D and survival variables

Data were obtained from the institutional electronic medical record system. Baseline serum concentration of 25(OH)D and all subsequent serum measurements were divided into quartiles for analysis defined as deficient (<20 ng/mL), insufficient (20–39 ng/mL), sufficient (40–59 ng/mL), and optimal (≥60 ng/mL). Quartiles were determined based on cutoffs used in literature in addition to nutritionist and naturopathic physician recommendations from our facility for our patient population [3, 11, 19, 24]. Measured as days from diagnosis to progression of disease, TTP (defined as change in treatment supported by objective evidence of progression of disease or death in the absence of documented disease progression) was recorded for each patient. Overall survival, measured as days from diagnosis to date of death, was also recorded for each patient. The date of last patient contact was used to represent TTP and/or OS for patients without documented progression, date of death, or for those patients still living. Statistical survival analyses censored patients fitting these conditions.

Three variables known to influence serum levels of 25(OH)D include age, body mass index (BMI), and ethnicity. This study did not evaluate the effect of age on 25(OH)D, but did assess age at diagnosis. For this study, BMI was divided into quartiles: underweight (<18.5 kg/m^2^), healthy weight (18.5–24.9 kg/m^2^), overweight (25–29.9 kg/m^2^), and obese (≥30 kg/m^2^). Self-reported ethnicity was recorded for each patient.

### Statistical analysis

Statistical analyses were completed using the Statistical Analysis System (SAS). The initial analysis for each primary variable, TTP and OS, compared the 25(OH)D quartiles based on the use of Kaplan-Meier survival curves and the Log Rank test. Additionally, the vitamin D groups were compared after adjusting for important covariates, including number of prior cancer therapies, BMI, and ethnicity, using the Cox Proportional Hazard model. Patients lost to follow-up, and, therefore lacking a documented date of progression or death, were censored. Statistical significance was declared if the two-sided *p*-value was ≤ 0.05.

## Results

A total of 1222 patients with pancreatic cancer were seen during the defined time period, of which 1017 patients had at least one had recorded serum concentration of 25(OH)D. Out of the 1017 patients who had baseline vitamin D values, 627 had not received cancer directed therapy prior to admission at CTCA. Because prior treatment could affect nutritional status of a patient, only patients who had not received prior therapy were included for analysis.

**Fig. 1:**
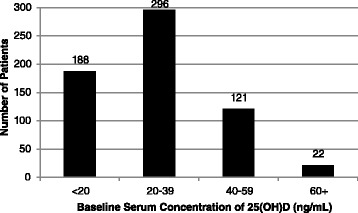
Distribution of baseline 25(OH)D levels among pancreatic cancer patients(n = 627). ng/mL: nanogram per milliliter

As summarized in Table 1, the majority of subjects were Caucasian and had stage IV pancreatic cancer. The average age at diagnosis was 57 (range: 31–82, SD:7.7). Gender was equally represented, and the majority of the samples were 25(OH)D insufficient. The patients who had a BMI of 25 or higher (33 %) primarily fell into the insufficient and deficient 25(OH)D quartiles. Even the majority of patients with a healthy BMI had insufficient BSC of 25(OH)D (48 %). Figure 1 depicts the frequency of patients per baseline 25(OH)D quartile. Of the patient characteristics analyzed, ethnicity (*p* < 0.0001) and BMI (*p* = 0.05) were statistically significantly associated with BSC of 25(OH)D.

**Table 1:** Distribution of Demographics Grouped by 25(OH)D Quartile

	Vitamin D Quartile						
	<20 ng/mL	20–39 ng/mL	40–59 ng/mL	60 + ng/mL	Total	25(OH)D	
Characteristic	No. patients (%)	No. patients (%)	No. patients (%)	No. patients (%)	No. patients (%)	Avg. ng/mL (SD)	*P*-value
Gender							
Male	105 (56)	167 (56)	63 (52)	7 (32)	342 (55)	28 (±14)	0.15
Female	83 (44)	129 (44)	58 (48)	15 (68)	285 (45)	30 (±17)	
Ethnicity							<0.0001
Caucasian	118 (25)	236 (50)	106 (22)	16 (3)	476 (76)	31 (±15)	
African American	57 (53)	36 (34)	11 (10)	3 (3)	107 (17)	22 (±15)	
Hispanic	0 (0)	7 (44)	0 (0)	1 (6)	16 (3)	22 (±14)	
Asian	0 (0)	4 (100)	0 (0)	0 (0)	4 (0.6)	30 (±6)	
Polynesian	0 (0)	0 (0)	1 (100)	0 (0)	1 (0.2)	48 (±0)	
Other*	5 (22)	5 (22)	3 (13)	2 (9)	23 (4)	30 (±16)	
Cancer Stage							0.36
1B	4 (50)	2 (25)	2 (25)	0 (0)	8 (1)	23 (±14)	
2A	13 (48)	9 (33)	4 (4)	1 (4)	27 (4)	24 (±16)	
2B	15 (31)	27 (56)	3 (6)	3 (6)	48 (8)	27 (±14)	
3	24 (28)	40 (47)	19 (22)	3 (3)	86 (14)	30 (±15)	
4	131(29)	216 (48)	92 (20)	15 (3)	454 (72)	29 (±16)	
Non-staged	1 (25)	2 (50)	1 (25)	0 (0)	4 (1)	28 (±11)	
BMI (kg/m^2^)							0.05
< 18.5	7 (27)	8 (31)	9 (35)	2 (8)	26 (4)	35 (±19)	
18.5–24.9	67 (25)	126 (48)	57 (22)	14 (5)	264 (42)	31 (±17)	
25–29.9	74 (36)	98 (47)	33 (16)	2 (1)	207 (33)	26 (±13)	
30+	40 (31)	64 (49)	22 (17)	4 (3)	130 (21)	28 (±14)	

*Other ethnicities are patients who did not identify with any ethnicity listed above

**Fig. 2:**
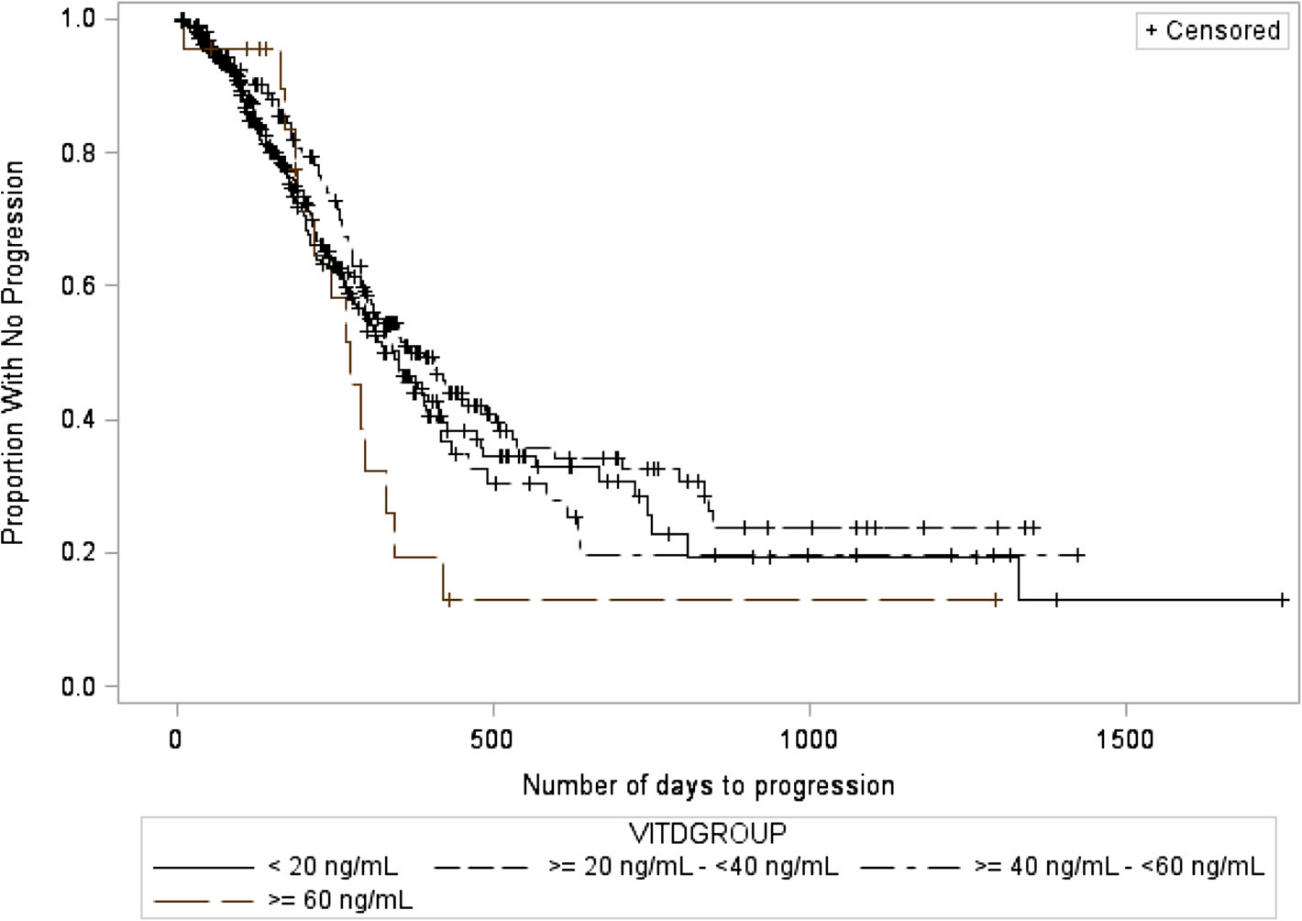
Kaplan-Meier survival curve depicting the association between baseline 25(OH)D quartile and time to disease progression (*p*=0.39)

Figure 2 depicts the average TTP per baseline 25(OH)D quartile. Kaplan-Meier survival analysis resulted in no statistically significant association between TTP and BSC of 25(OH)D (*p* = 0.39, deficient vs. insufficient quartiles HR = 1.1; 95 % CI = 0.9 to 1.5, deficient vs. sufficient quartiles HR = 1.1; 95 % CI = 0.8 to 1.5, deficient vs. optimal quartiles HR = 0.7; 95 % CI = 0.4 to 1.3, insufficient vs. sufficient quartiles HR = 0.9; 95 % CI = 0.7 to 1.3, insufficient vs. optimal quartiles HR = 0.6; 95 % CI = 0.4 to 1.1, sufficient vs. optimal quartiles HR = 0.7; 95 % CI = 0.4 to 1.2). For the progression analysis, 317 observations (52 %) were censored given that a documented date of disease progression or death was not available. Other variables of interest analyzed using a Type III ANOVA test, including ethnicity (*p* = 0.35, HR = 1.2; 95 % CI = 0.9 to 1.70) and BMI (*p* = 0.70, HR = 1.0; 95 % CI = 0.98 to 1.0), were not statistically significantly associated with TTP.

**Fig. 3:**
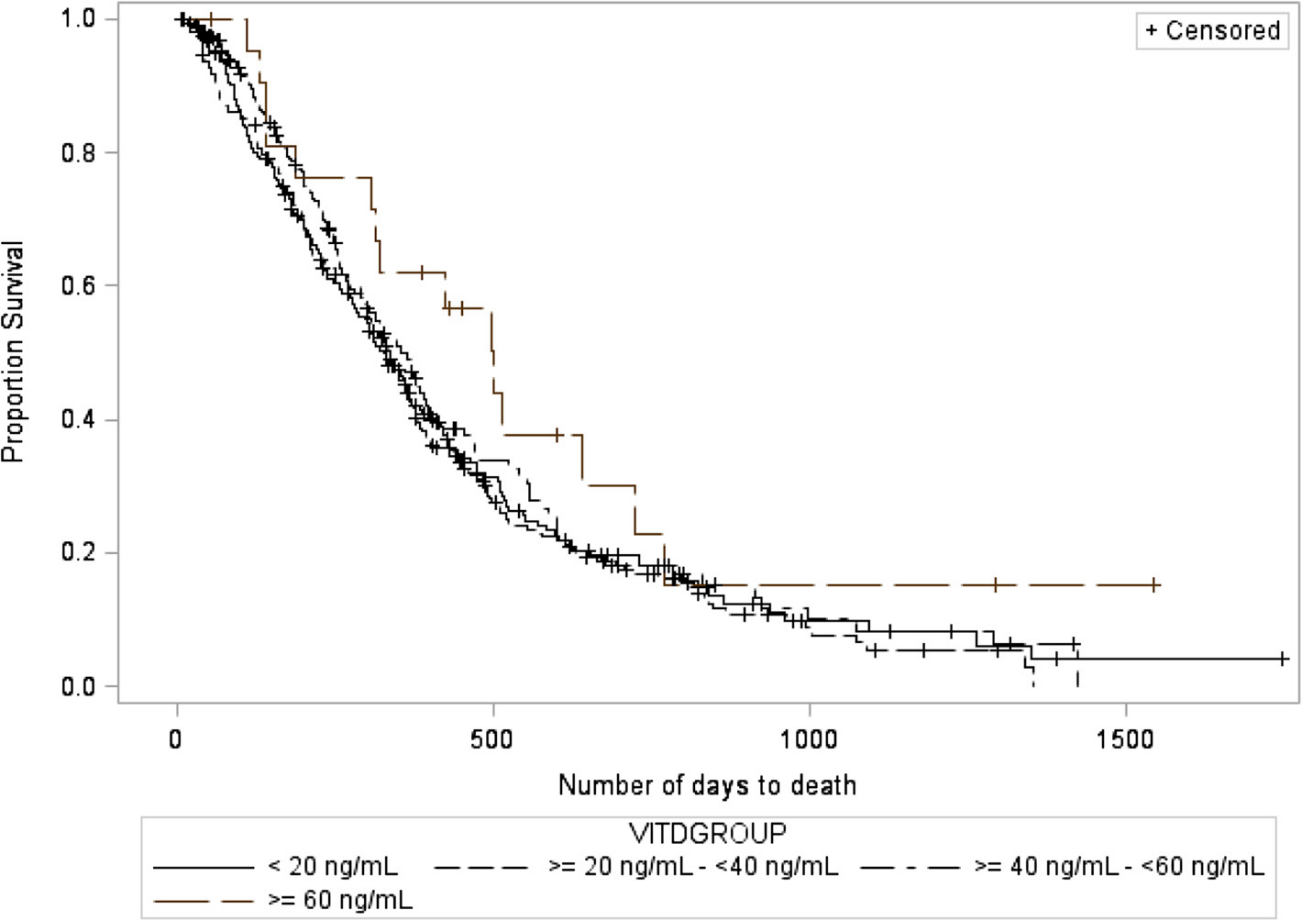
Kaplan-Meier survival curve depicting the association between baseline 25(OH)D quartile and overall survival in pancreatic cancer patients (*p*=0.37)

At the time of data collection, 480 (76 %) of the study participants had expired. Statistical analysis of average OS per baseline 25(OH)D quartile, depicted in Figure 3, did not result in a significant relationship between the two variables (*p* = 0.37, deficient vs. insufficient quartiles HR = 1.0; 95 % CI = 0.8 to 1.2, deficient vs. sufficient quartiles HR = 1.0; 95 % CI = 0.8 to 1.4, deficient vs. optimal quartiles HR = 1.6; 95 % CI = 0.9 to 2.7, insufficient vs. sufficient quartiles HR = 1.1; 95 % CI = 0.8 to 1.3, insufficient vs. optimal quartiles HR = 1.6; 95 % CI = 0.9 to 2.7, sufficient vs. optimal quartiles HR = 1.5; 95 % CI = 0.9 to 2.6). For the overall the survival analysis, 143 observations (23 %) were censored given that a documented date of death was not provided. A Type III ANOVA revealed that ethnicity (*p* = 0.79, HR = 1.1; 95 % CI = 0.8 to 1.4) and baseline BMI (*p* = 0.84, HR = 1.0; 95 % CI = 0.98 to 1.0) were not statistically significantly associated with OS.

Thus, the statistical results of this study showed both ethnicity and BMI impact BSC of 25(OH)D, and there is no statistically significant relationship between BSC of 25(OH)D and TTP or OS.

## Discussion

This analysis is one of the first to analyze the association between BSC of 25(OH)D and both TTP and OS in patients with pancreatic cancer. The results of this study showed that BSC of 25(OH)D is not statistically significantly associated with either TTP or OS. Therefore, BSC of 25(OH)D does not appear to be indicative of prognosis in pancreatic cancer patients.

Despite literature noting the protective properties of 25(OH)D and cancer development, the current data does not support an association between BSC of 25(OH)D and disease progression in patients with pancreatic cancer. Although graphical depiction suggests optimal BSC of 25(OH)D is associated with greatest OS, the same quartile experiences the most rapid TTP. Further research specifically aimed at understanding the role of 25(OH)D during the course of disease in pancreatic cancer, is needed to identify the presence or absence of such protective properties against disease progression.

In a recent correlative randomized trial, serum 25(OH)D of 256 pancreatic cancer patients was analyzed for associations with TTP and OS. Similar to our findings, the results showed no significant association between 25(OH)D and either variable (TTP *p* = 0.6, OS *p* = 0.95). However, that study did identify a statistically significant relationship between ethnicity and 25(OH)D, placing emphasis on the primary endogenous metabolism of vitamin D following absorption by the skin [19]. Because darker skin pigments absorb less UV light from the sun, darker-skinned individuals will likely have lower serum concentration of vitamin D than lighter-skinned individuals. Similar to existing literature, our study identified a statistically significant relationship between ethnicity and BSC of 25(OH)D (*p* < 0.0001).

Body Mass Index (BMI) is another factor cited in the literature as having an inverse influence on serum vitamin D [25]. Our study demonstrated a statistically significant relationship between BMI and BSC of vitamin D (*p*=0.05). In a study evaluating serum concentration of 25(OH)D in healthy individuals compared to obese individuals, researchers found that 25(OH)D was inversely correlated with BMI (*p* = 0.007) [26]. While basal rates of serum 25(OH)D did not differ immediately post radiation, a statistically significant difference emerged between obese and healthy individuals 24 h after exposure. Similarly, there was a significant association between BMI and peak 25(OH)D levels in an oral challenge [26]. Our study only observed 25(OH)D and BMI at baseline, a limitation that may have prevented identifying a dynamic association between changes in BMI with changes in 25(OH)D over time. Since subcutaneous fat is a deposition site for vitamin D, overweight and obese individuals may sequester vitamin D from the blood and store it in a non-readily available form, causing a prevalence of vitamin D deficiency among individuals with increased BMIs [23]. The association between BMI and 25(OH)D identified in the current study indicates an important area for future research.

The strength of the current study lies in the large sample size included for analysis; however, the design was retrospective in nature. Although it provides insight into possible associations between vitamin D and pancreatic cancer, a prospective study is subject to less bias and possible confounders. The retrospective nature of the study limits the ability to account for initiation of and compliance to vitamin D supplementation. Some patients expired prior to documentation of progression of disease. For these patients, date of expiration was used for both OS and TTP as death was considered disease progression. Given our patient population travels from many states across the country, as well as internationally, it was not feasible to assess the season during which blood was drawn (and, therefore relative sun exposure) to determine potential effects on serum25(OH)D.

Our analysis suggests that vitamin D is not significantly associated with rates of disease progression or overall survival in an advanced pancreatic cancer population; however, the limitations of a retrospective and the inconclusive reports of this topic in the literature suggest more prospective trials are needed to further define potential associations between vitamin D and pancreatic cancer progression. The most recent literature evaluating the effect of vitamin D on gastrointestinal disease, including pancreatic cancer, continues to identify the inconsistencies and contradictions of published findings, as well as the need for a prospective human interventional trial [27]. Furthermore, research is moving towards analysis of vitamin D analogs and their potential to activate the desirable anti-inflammatory, immunomodulatory, and apoptotic effects while avoiding the homeostatic effects on calcium and phosphorous levels. Analogs have also been cited to synergistically enhance the desired effects of chemotherapy when used in combination [28, 29]. Future research should prospectively document the variables assessed in this current study to best define associations between BSC of 25(OH)D and OS and TTP.

## Conclusion

This study did not identify a significant association between 25(OH)D and overall survival or time to progression in patients with pancreatic cancer. Future prospective studies should explore potential relationships between 25(OH)D and OS and TTP in pancreatic cancer. Additional studies may also apply analysis to changes in serum 25(OH)D in association with changes in other variables, such as BMI.

## Abbreviations

ANOVA: analysis of variance
BMI: body mass index
BSC: baseline serum concentration
CTCA: Cancer Treatment Centers of America
IRB: institutional review board
ng/mL: nanogram per milliliter
OS: overall survival
TTP: time to progression
UVB: ultraviolet B-rays
VDR: vitamin D receptor
